# Electrically‐Switchable Gain in Optically Pumped CsPbBr_3_ Lasers With Low Threshold at Nanosecond Pumping

**DOI:** 10.1002/smll.202411935

**Published:** 2025-02-24

**Authors:** Yang Li, Shangpu Liu, Thomas Feeney, Julie Roger, Mohammad Gholipoor, Hang Hu, Dewei Zhao, Ian Howard, Felix Deschler, Uli Lemmer, Ulrich W. Paetzold

**Affiliations:** ^1^ College of Materials Science and Engineering & Engineering Research Center of Alternative Energy Materials and Devices Ministry of Education Sichuan University Chengdu 610065 China; ^2^ Institute of Microstructure Technology Karlsruhe Institute of Technology Hermann‐von‐Helmholtz‐Platz 1 76344 Eggenstein‐Leopoldshafen Germany; ^3^ Light Technology Institute Karlsruhe Institute of Technology Engesserstrasse 13 76131 Karlsruhe Germany; ^4^ Physikalisch‐Chemisches Institut Universität Heidelberg Im Neuenheimer Feld 229 69120 Heidelberg Germany

**Keywords:** VCSEL, continuous‐wave, electrically injection lasing, ion migration, perovskite

## Abstract

Metal halide perovskites hold promise for nonepitaxial laser diodes, yet, continuous‐wave (CW) optically pumped (photonic) lasing in CsPbBr_3_ remains elusive despite its superior thermal‐ and photo‐stability among the perovskite family. This work reports on CsPbBr_3_ vertical cavity surface emitting lasers with low lasing thresholds (1.3 µJ cm^−^^2^) at nanosecond pumping and remarkable lasing stability. Furthermore, the electrically switchable gain is achieved in CsPbBr_3_ electrically assisted optically pumped laser (EAOPL) devices by leveraging ion migration. Applying a small positive DC voltage to the EAOPL device significantly reduces the lasing threshold under nanosecond laser excitation and enhances the cavity mode intensity at CW laser excitation. These findings present a novel strategy, combining a small DC voltage with an electrical pulse, for exploring electrical injection lasing in CsPbBr_3_ perovskites.

## Graphical abstract

This work introduces a low‐threshold, nanosecond‐pumped CsPbBr_3_ laser fabricated through a hot‐embossing lamination process. In an electrically assisted optically pumped laser device, it is demonstrated that an electric bias can enhance and switch the optical gain of the CsPbBr_3_ laser via field‐induced ion migration.

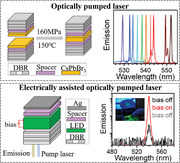

## 1 Introduction

Halide perovskite semiconductors have emerged as a promising candidate for a new class of versatile, non‐epitaxially grown semiconductor laser diodes^[^ ^1^, ^2^ ^]^ given their exceptional optoelectronic properties, including high absorption coefficients, high carrier mobilities, and low‐cost solution processability. Continuous wave (CW) optically pump lasing is widely considered a crucial milestone toward achieving an electrically driven laser diode. So far, perovskite semiconductors of a wide range of compositions such as MAPbI_3_,^[^ ^3^, ^4^ ^]^ (Cs, FA, MA)Pb(I,Br)_3_,^[^ ^5^, ^6^ ^]^ CsPb(I_,_Br)_3_‐Zn(Ac)_2_^[^ ^7^ ^]^ and quasi‐2D perovskites^[^ ^8^, ^9^ ^]^ have demonstrated CW optically pumped (photonic) lasing or amplified spontaneous emission (ASE). Surprisingly, to date, CsPbBr_3_ thin films which are one of the most stable perovskite materials with suitable bandgap in the optical spectrum not yet demonstrated CW (photonic) lasing. Although CW ASE (with an extremely high threshold) has been reported in CsPbBr_3_ single crystals,^[^ ^10^ ^]^ this has not translated to thin films. Meanwhile, CW exciton‐polariton lasing has been observed in CsPbBr_3_ nanowires and nanoribbons.^[^ ^11^, ^12^ ^]^ However, their application to commercial products faces significant hurdles. Given its excellent thermal and photochemical stability,^[^ ^13^, ^14^ ^]^ CsPbBr_3_ remains a highly attractive material for electrically injection lasing. This work presents a low‐threshold, stable vertical cavity surface emitting laser (VCSEL) based on CsPbBr_3_ bulk films and investigates the factors limiting CW lasing.

Beyond CW optically pumped lasing, electrically assisted optically pumped laser (EAOPL), utilizing the electrical‐optical co‐pumping scheme,^[^ ^6^, ^15^, ^16^, ^17^ ^]^ provides a valuable platform for advancing perovskite semiconductors toward electrically injection lasing. Recent breakthroughs^[^ ^6^, ^16^ ^]^ have demonstrated that intense carrier injection into perovskite light‐emitting diodes (PeLEDs) contributes to optical gain. These studies, focusing on red‐emitting PeLEDs, achieved a 13% reduction in optically pumped ASE threshold at 77 K^[^ ^6^ ^]^ and a 24% reduction in optically pumped lasing threshold at 230 K,^[^ ^16^ ^]^ respectively. Prior to these, we reported a substantially higher 300% reduction in the optically pumped ASE threshold of a CsPbBr_3_ LED at room temperature using a small positive DC voltage.^[^ ^15^ ^]^ This enhancement was attributed to the field‐induced migration of ionic defects, which regulated photogenerated charge carriers' radiative and nonradiative recombination, rather than direct electrical injection by DC voltage.^[^ ^15^ ^]^ Building upon this, we integrated a CsPbBr_3_ LED with an optical cavity to realize an EAOPL device.

This paper reports on a CsPbBr_3_ VCSEL with a remarkably low optically pumped lasing threshold of 1.3 µJ cm^−2^ under nanosecond pulsed laser excitation. This is the lowest lasing threshold reported to date for CsPbBr_3_ thin films. Meanwhile, the VCSEL exhibits remarkable operational stability of laser emission owing to the inherent stability of the CsPbBr_3_ gain medium and device encapsulation via lamination.^[^ ^18^, ^19^, ^20^, ^21^ ^]^ Furthermore, we demonstrate a two‐fold reduction in the optically pumped lasing threshold of an EAOPL device under nanosecond laser excitation by applying a 4 V positive DC bias. This DC bias also significantly enhances cavity mode intensity under CW laser excitation.

## 2 Results and Discussion

### 2.1 Optically Pumped CsPbBr_3_ Laser

**Figure 1:**
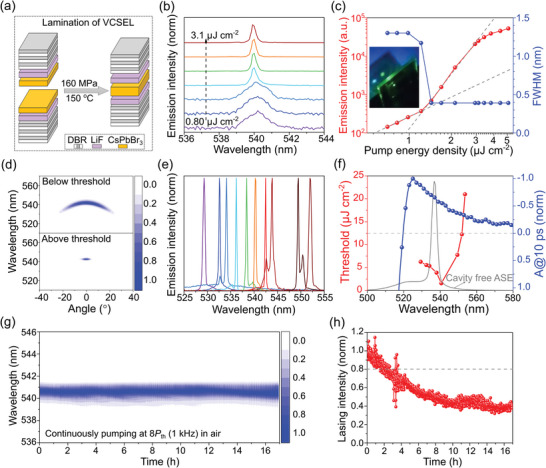
Fabrication and characterization of CsPbBr_3_ perovskite VCSEL. a) Schematic illustration of the perovskite VCSEL fabricated using a lamination process. b) Evolution of emission spectra with increasing pump energy densities (355 nm, 1 ns, 1 kHz). The emission spectra were normalized and shifted vertically by a constant offset for clarity. c) Emission intensity and FWHM as a function of pump energy density. Linear fits were shown below and above the lasing threshold (*P*th). The inset showed the far‐field image of the VCSEL emission (green) and the pump laser (blue, incidence angle: 45°) at ≈3 cm. d) Angle‐resolved emission spectra of VCSEL below and above the lasing threshold. e) Normalized lasing spectra for VCSELs with different LiF spacer thicknesses, i.e., lasing modes. f) Lasing threshold and transient gain spectrum as a function of emission wavelength and cavity‐free ASE spectrum. g) Temporal evolution of normalized lasing spectra for a VCSEL pumped at a constant power of 8*P*_th_ (pump laser power fluctuation: ≤ 2%, repetition rate: 1 kHz) in air. h) Normalized lasing intensities as a function of time.

To fabricate the VCSEL, commercially distributed Brag reflectors (DBRs) with an average reflectance > 99% in the relevant spectral range (500‒600 nm) were adopted as the reflectors. Thin films of LiF and solution‐processed CsPbBr_3_ were deposited on the DBRs as cavity spacers and gain media, respectively. Details regarding the CsPbBr_3_ thin films can be found in our previous publication.^[^ ^21^, ^22^ ^]^ As shown in **Figure** **1**a, two such stacks were laminated at 160 MPa and 150 °C for 5 min to complete the VCSEL. Due to elevated temperature and pressure, the CsPbBr_3_ thin films recrystallized and coalesced, enabling the bonding of two rigid DBR substrates.^[^ ^18^ ^]^ The transmittance spectra of VCSEL exhibited a clear resonance within the stopband of DBR (Figure S1a, Supporting Information), demonstrating the formation of a uniform cavity by the lamination process. By varying the thickness of the LiF spacer, the cavity length and cavity mode could be precisely tuned (Figure S1b, Supporting Information).

The optically pumped lasing performance of laminated VCSEL was investigated by excitation with a 355 nm pulsed laser (1 ns, 1 kHz). Unless otherwise specified, all measurements were conducted in ambient conditions at room temperature with an incident angle of 45° for the pump laser. Along with increasing pump energy densities from 0.8 to 3.1 µJ cm^−2^ (Figure 1b), a sudden blueshift in the emission peak wavelength accompanied by a narrowing of the emission spectrum was observed, suggesting the onset of lasing. Lasing was further verified by the corresponding light‐in‐light‐out (L‐L) curves and full width at half maxima (FWHM) analysis (see Figure 1c). The L‐L characteristic shows a clear transition from linear to superlinear regime with slope (in log‐log scale) increasing from 1.7 to 4.8. Concurrently, FWHM decreases from 1.2 to 0.39 nm. This suggests lasing with a threshold (*P*_th_) of 1.3 µJ cm^−2^ (carrier density of 2.6 × 10^17^ cm^−3^), representing the lowest lasing threshold reported to date for CsPbBr_3_ thin films (see Table S1, Supporting Information). The best Q factor of the VCSELs was estimated as 1384. As illustrated in the inset of Figure 1c, the far‐field pattern of the lasing beam displayed a small‐diameter, circular green spot emitted from the surface normal at a detection distance of ≈3 cm, as expected from VCSEL. Moreover, this circular beam spot remained visible at a distance of ≈20 cm from the VCSEL surface (Video S1, Supplementary), indicating beam coherence with small divergence, which was further examined using angle‐resolved emission measurement. As shown in Figure 1d, when increasing pump energy density above the lasing threshold significantly reduced the output beam divergence, leading to a well‐collimated lasing beam. Notably, angle‐resolved emission spectra revealed the cavity photon dispersion rather than exciton‐polariton dispersion (Figure S2, Supporting Information). Furthermore, free carriers rather than excitons were responsible for radiative recombination (at near transparency carrier densities) in our CsPbBr_3_ thin film.^[^ ^21^ ^]^ Therefore, even though our threshold carrier density is close to (or below) the reported values (1.8‒4.7 × 10^17^ cm^−3^)^[^ ^23^ ^]^ of Mott density, the laser emission from our CsPbBr_3_ VCSEL was attributed solely to photonic lasing.

By varying the thickness of LiF thin film, the lasing mode was tuned across a broad spectral range from 529 to 552 nm (see Figure 1e), revealing a substantial optical gain bandwidth^[^ ^24^ ^]^ of 23 nm. The laser linewidth was in the range of 0.2‒0.6 nm and the spectral resolution of the spectrometer was 0.13 nm. The dependence of the lasing threshold and wavelength was depicted in Figure 1f (red curve, referred to as threshold spectrum), showing the lowest threshold at a lasing wavelength of 541 nm. Employing femtosecond transient absorption (fs‐TA) pump‐probe experiment (Figure S3, Supporting Information), the transient gain spectrum (blue curve in Figure 1f) was derived based on the negative absorption signal^[^ ^25^, ^26^ ^]^ of CsPbBr_3_ thin film upon excitation with a 260 fs pump laser (450 nm). Following the fs laser excitation, the optical gain in CsPbBr_3_ thin film emerged within a few picoseconds and persisted for over 100 ps (Figure S3, Supporting Information). In agreement with the threshold spectrum (Figure 1f), the gain spectrum covered a wider spectral range from 520 to 560 nm than the ASE band (grey curve in Figure 1f), which rationalized the lasing modes (Figure 1e,f) outside the ASE band. We also observe the distinctions among the threshold spectrum, gain spectrum, and ASE spectrum, with a comprehensive discussion provided in Figure S3 (Supporting Information).

To assess lasing stability, lasing spectra were monitored at a constant pump power of ≈8*P*th in ambient conditions for several hours (pump laser: 355 nm, 1 ns, 1 kHz), as shown in Figure 1g,h. The T80 lifetime at 8*P*th was estimated to be 2 h. Despite the harsh conditions, laser emission intensity sustained 40% of its initial value after continuously pumping the VCSEL at 8*P*th for 17 h in ambient conditions. These results demonstrated the good operational stability of the VCSEL, which could be attributed to the inherent stability of the CsPbBr_3_ perovskite and the device encapsulation by the lamination process.

### 2.2 Advancing Optically Pumped CsPbBr_3_ Lasing from Pulsed to CW Operation

**Figure 2:**
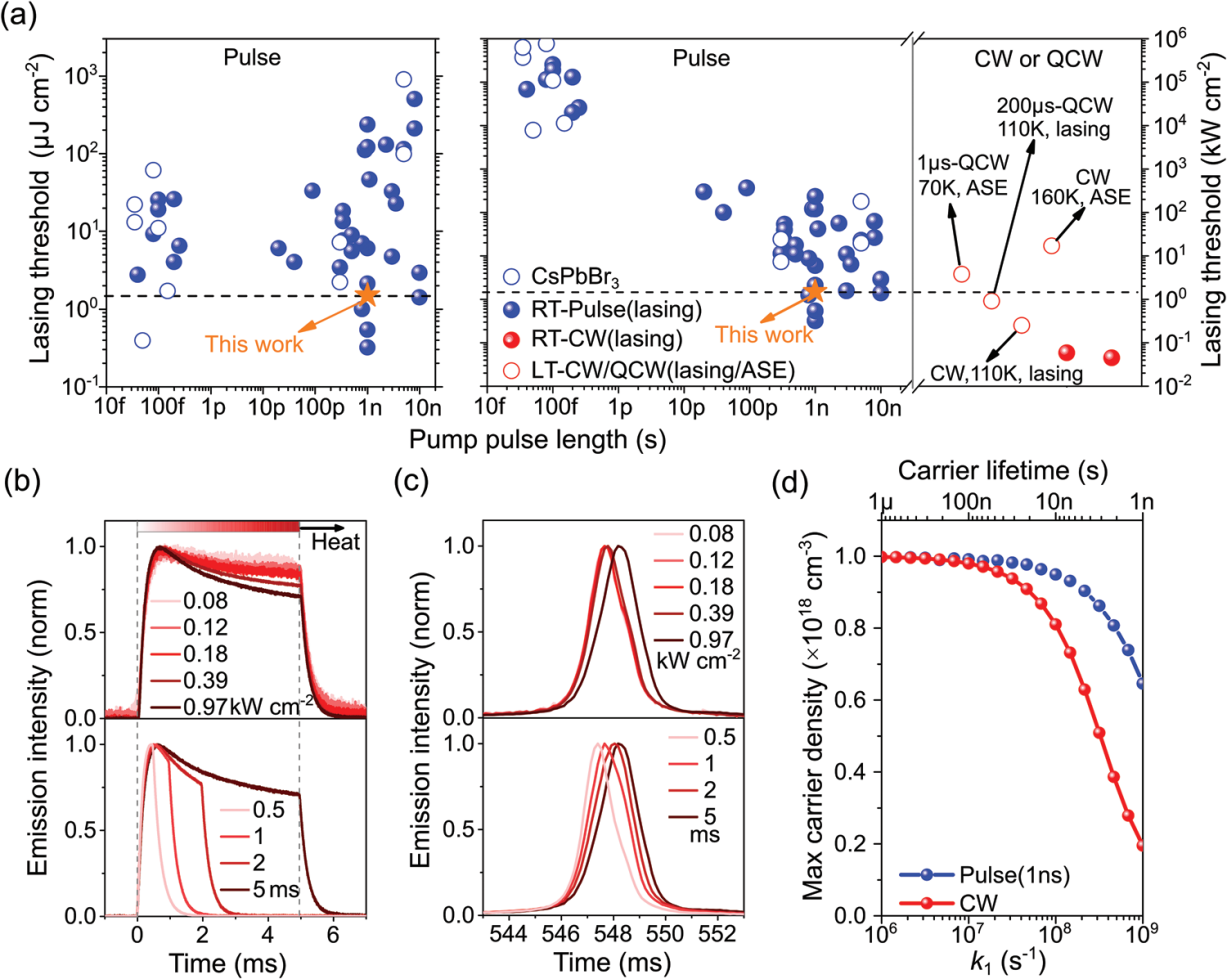
Optically pumped lasing from pulsed to CW operation. a) Comparison of reported thresholds in energy density (left) and power density (right) as a function of pump pulse length. Power density (kW cm^−2^) was calculated by dividing energy density (µJ cm^−2^) by corresponding pump pulse length. For QCW/CW excitation, reported thresholds (ASE and lasing) at low temperatures (LT) were included. The blue open circles represented the lasing threshold of CsPbBr_3_ thin films. The yellow star denoted the lasing threshold of this study. b) Normalized transient emission intensity and c) normalized steady‐state emission spectra versus pump power densities (pulse length: 5 ms) and pump pulse length (power density: 1 kW cm^−2^). d) Simulated maximum carrier densities under pulsed (1 ns) and CW operation as a function of 1st order rate coefficient (*k*_1_). The simulation was performed by solving rate equation d*n*/d*t* = *G*(*t*) ‒ *k*_1_ *n* ‒ *k*_2_ *n* ^2^ ‒ *k*_3_ *n* ^3^, where *k*_2_ (10^−10^ cm^3^ s^−1^) and *k*_3_ (10^−28^ cm^6^ s^−1^) are the 2^nd^ and 3^rd^ order rate coefficients, respectively, and *G* is the pump rate. The pump flux and pump rate were fixed as 1.06 × 10^18^ cm^−3^ and 2 × 10^26^ cm^−3^ s^−1^ for pulsed and CW operation, respectively, corresponding to a peak carrier density of 10^18^ cm^−3^ for small *k*_1_ value.

**Figure** **2**a illustrates the reported lasing threshold of halide perovskite lasers, primarily focusing on VCSEL and DFB  laser, and lasers with a natural cavity, such as whispering gallery mode lasers and nanorods lasers, were excluded for clarity. To facilitate comparison between pulsed and QCW/CW pumping regimes, lasing thresholds in power density (kW cm^−2^) were also shown for pulsed pumping regimes, and more details can be found in Table S1 (Supporting Information). Interestingly, the power density thresholds decreased with increasing pump pulse length. This was surprising at first sight, as lasing under QCW/CW operation appeared much more straightforward compared to ultrashort pulse excitation. This can be explained by the carrier steady‐state condition (see Figure S4, Supporting Information): when pump pulse length exceeds carrier lifetime, pump power rather than pump energy determines the lasing threshold; otherwise, pump energy matters.

Among all reported perovskite lasers, our laminated VCSEL exhibited an outstanding lasing threshold (Figure 2a). These seeded interest in exploring room temperature CW lasing of our VCSEL, whose lasing threshold could be estimated from power densities lasing threshold at nanosecond pumping as ≈1 kW cm^−2^. We note that the CW lasing threshold can be better approximated by the ratio of the threshold fluence measured using ultrafast excitation and the gain lifetime.^[^ ^27^ ^]^ To minimize the heating effect, a 405 nm CW laser diode operated at QCW mode with a 1 ms pulse was utilized as the pump source. Although a few laser‐like features under QCW laser excitation were apparent (Figure S5a–c, Supporting Information), neither output power nor FHWM exhibited a clear threshold behavior across a wide range of pump power densities (0.1–300 kW cm^−2^). Angle‐resolved emission spectra (Figure S5d, Supporting Information) further ruled out the thresholdless lasing behavior,^[^ ^28^, ^29^, ^30^ ^]^ suggesting the absence of room temperature CW lasing in our laminated VCSEL.

To elucidate the factors hindering CW lasing in our CsPbBr_3_ perovskite, the transient emission intensity of the VCSEL within a long pump pulse (5 ms) of QCW laser was monitored. Under the steady‐state condition of photo‐carriers, if the laser power remains the same within the pump pulse length, emission intensity is expected to remain unchanged in the same time scale (see Figure S6, Supporting Information). However, the emission intensity dropped within the pump pulse length, as shown in Figure 2b. Moreover, the attenuation of emission intensity within the pump pulse length became more pronounced with increasing pump power densities (Figure 2b upper panel) or pump pulse lengths (Figure 2b lower panel), which were accompanied by a redshift in the emission spectra (Figure 2c). Apparently, the greater the input energy (higher power or longer pulse), the greater the change in transient and steady‐state emission of VCSEL. These results were a clear indication of the pump‐induced heating effect. Notably, unlike the emission peak blueshift and high energy spectral broadening (Figure S7, Supporting Information) observed in perovskite thin films due to carrier heating,^[^ ^31^, ^32^ ^]^ the emission peak redshift (cavity mode: m*λ* = 2*nL*) in our VCSEL is attributed to the changes in refractive index^[^ ^33^ ^]^ and cavity length caused by material heating.

To mitigate the pump‐induced heating effect, the laminated VCSEL was operated at a cryonic temperature of 80 K. However, no clear signature of CW lasing was observed (Figure S8, Supporting Information), suggesting that the heating effect is not the sole limiting factor. The other plausible factor is the high density of trap states,^[^ ^31^ ^]^ which is suggested by the short PL lifetime and the low PLQY of this CsPbBr_3_ thin film.^[^ ^22^ ^]^ Under steady‐state conditions, 1^st^ order trap‐related nonradiative recombination leads to continuous carrier leakage from the carrier reservoir generated by CW laser. Conversely, for a short excitation pulse (≤1 ns), 1^st^ order nonradiative recombination is negligible, allowing for the generation of high carrier densities. This could be visualized by comparing the impact of 1^st^ order rate coefficient (*k*_1_) on the maximum carrier densities under pulsed and CW operation, as illustrated in Figure 2d. To some extent, this explained why our laminated VCSEL could achieve a low lasing threshold under 1 ns laser excitation, but failed to achieve CW lasing. In this regard, effective trap passivation and thermal management for CsPbBr_3_ thin film needs to be implemented. Besides, the 2^nd^ order radiative rate is also crucial to the realization of CW lasing, as it affects the population inversion and stimulates emission rate in opposite directions (Figure S9, Supporting Information).

### 2.3 Electrically Assisted Optically Pumped CsPbBr_3_ Laser

**Figure 3:**
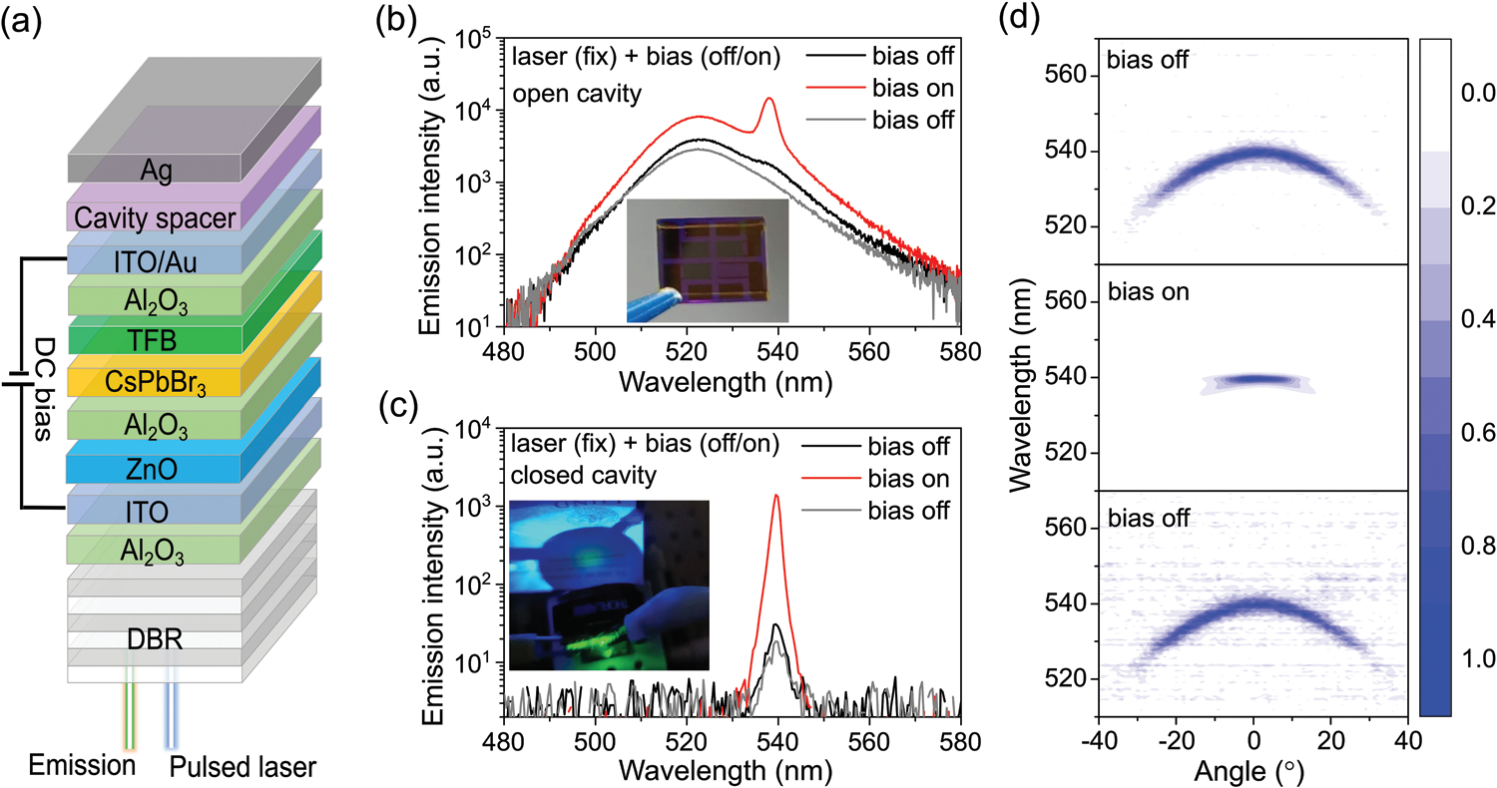
Electrically switchable gain in an EAOPL device. a) Device architecture of the perovskite EAOPL device. The 1 ns pulsed laser (355 nm) was incident perpendicular to the DBR back surface. b, c) Switching on and off optically pumped b) ASE, and c) lasing by applying a positive 4 V DC bias while maintaining the pulsed laser power at 0.6*P*_th_. The emission of these devices stabilized ≈10 s after switching bias. The inset in b) shows the photograph of the device with one open cavity cell (without Ag) and three closed cavity cells (with Ag). The inset in c) shows the green lasing beam spot at ≈3 cm in front of the device, and a long pass filter was placed in front to filter out the pump laser emission. d) Comparison of angle‐resolved emission spectra for closed cavity device between switching on and off positive 4 V DC bias. The power of the pulsed laser was maintained at 0.6*P*_t__h_.

Having demonstrated the excellent and stable performance of our optically pumped CsPbBr_3_ lasers, we further demonstrated that the laser performance could be enhanced and modulated by an electric bias in an EAOPL device (see **Figure** **3**a; Figure S10, Supporting Information). The EAOPL device was fabricated by integrating a CsPbBr_3_ LED with an optical cavity. Since the high temperatures required for lamination were incompatible with LED fabrication, the DBR‐Ag cavity configuration was employed instead of the DBR‐DBR structure, leading to a lower cavity Q factor. Prior to the LED fabrication, a 150 nm thick Al_2_O_3_ thin film was deposited via ALD on top of DBR to protect it from subsequent ITO sputtering and facilitate heat dissipation. The LED's architecture consisted of two ITO (10 nm) electrodes, a ZnO electron transporting layer, a TFB hole transporting layer, and a thin CsPbBr_3_ film. To compensate for the lower conductivity of thin ITO electrodes, 200 nm Au grids (0.2 mm width) were deposited on top. Further details regarding the optimization of the LED architecture can be found in our previous work.^[^ ^14^ ^]^ Besides, a LiF thin film served as a cavity spacer to tune the cavity mode. It should be noted that the lasing threshold of this EAOPL device under purely optical pumping increased by ≈20 times compared to laminated VCSEL, which could be attributed to 1) the higher ASE threshold^[^ ^15^ ^]^ of CsPbBr_3_ LED than that of CsPbBr_3_ thin film due to increased interface losses and electrodes absorption, and 2) higher cavity loss associated with DBR‐Ag resonator compared to the DBR‐DBR resonator.

The EAOPL was pumped by a 1 ns pulsed laser (355 nm) at a positive 4 V DC bias. With the pulsed laser power fixed at 0.6*P*_th_ (*P*_th_ represents the purely optically pumped threshold of the corresponding device), the emission spectra were recorded while switching the DC bias between 0 V and 4 V (Figure 3b,c). To investigate the impact of the optical cavity, devices with and without an Ag reflector were fabricated on a single substrate by shadowing one of the sub‐cells during Ag deposition (inset of Figure 3b). For the open cavity device (Figure 3c), applying a 4 V bias enabled ASE at a pump power of 0.6*P*_th_ and increased the PL intensity by a factor of 3. This enhancement was accompanied by an increase in PL lifetime (Figure S9, Supporting Information), which agreed with our previous findings regarding field‐induced migration of ionic defects.^[^ ^14^ ^]^ For the closed cavity counterpart (Figure 3c), a significantly greater emission intensity enhancement (≈50 times) was observed upon applying the 4 V bias, along with a spectral narrowing (from 3.4 to 1.5 nm) and the emergence of a green circular emission spot (inset of Figure 3c). To rule out the influence of ASE, which does not affect beam coherence (Figure S10, Supporting Information), angle‐resolved emission spectra of this EAOPL device were measured (Figure 3d). A clear reduction in beam divergence was evident when 4 V DC bias was applied in conjunction with the pulsed laser at 0.6*P*_th_. The decrease in emission linewidth along with the increase in beam coherence is a strong indication of lasing (539 nm) from the CsPbBr_3_ EAOPL device. Importantly, these results demonstrate the excellent electrical switching capabilities of the optically pumped laser. We found this switching behavior was highly repeatable, as visualized via the appearance and disappearance of the green lasing spot upon toggling the DC bias on and off (with pulsed laser power fixed at 0.6*P*_th_), as shown in Video S2 (Supplementary Video2). By adding a 4 V DC bias, the optically pump lasing threshold of the CsPbBr_3_ EAOPL device was reduced by a factor of 2. A higher voltage than 4 V did not further reduce the lasing threshold but did accelerate the response of lasing/ASE/PL to DC bias as the ions were expected to move faster.^[^ ^15^ ^]^

To assess the impact of DC bias under QCW operation, the EAOPL device was pumped with a QCW laser (1 ms) at a positive 4 V DC bias. Similar to the nanosecond laser excitation, the 4 V bias enhanced emission intensity (at 539 nm) generated by QCW laser for ≈4 times with high repeatability (Figure S11, Supporting Information). However, both L‐L characteristics and FWHM exhibited thresholdless behavior. Hence, while DC bias enhanced the cavity mode intensity under CW excitation, but did not result in CW lasing at this stage. Although electric bias could mitigate the trap effects under CW operation by removing and immobilizing mobile defects, the significantly higher lasing threshold of the EAOPL device compared to the laminate VCSEL counteracted this positive effect from electrical bias. In this regard, reducing the purely optically pumped threshold by engineering LED and microcavity (Q factor: 1380 for VCSEL and 360 for EAOPL device, as shown in Table S2, Supporting Information) will be crucial for achieving CW lasing in the CsPbBr_3_ EAOPL device. Importantly, our investigations into the EAOPL device demonstrate a general strategy of utilizing a small electric bias to mitigate the detrimental effect of mobile ionic defects on lasting performance. Given the ionic nature of halide perovskite semiconductors, this strategy holds significant promise, particularly for the study of electrically injection lasing. Moreover, it is imperative to focus on enhancing the electrical properties and thermal management of the current EAOPL device to achieve this objective.

## 3 Conclusion

This work demonstrated a low‐threshold optically pumped CsPbBr_3_ vertical‐cavity surface‐emitting laser (VCSEL) fabricated via a facile lamination technique. Under nanosecond pulsed excitation, the VCSEL exhibited a remarkably low lasing threshold of 1.3 µJ cm^−2^, a broad gain bandwidth of 23 nm, excellent beam coherence, and impressive operational stability with a T_80_ of 2 h (pumped at 8*P*_th_ in the air). Investigations into the limitations of CW lasing at room temperature revealed the detrimental effects of pump‐induced heating and high trap state densities within the CsPbBr_3_ perovskite. Furthermore, we introduced an electrically assisted optically pumped laser (EAOPL) device, showcasing the modulation of CsPbBr_3_ lasing through the application of a positive DC bias. A 4 V bias effectively reduced the optically pumped lasing threshold under nanosecond excitation by a factor of 2. Moreover, the electric bias enhanced the cavity mode intensity under CW excitation by ≈4 times. These findings represent a significant advancement toward the realization of both CW optically pumped lasing and electrically injected lasing in CsPbBr_3_ perovskite, paving the way for the development of efficient and versatile perovskite‐based lasers.

## 4 Experimental Section

### Materials

CsPbBr_3_ (>98.0%) was purchased from TCI. n‐Butylammonium bromide (BABr, 98%), DMSO (>99.0%), LiF, zinc acetate dihydrate (>99.995%), and ethanolamine (>99.5%) were purchased from Sigma Aldrich. 18‐Crown‐6 (>98%) was purchased from Alfa Aesar. Poly[(9,9‐dioctylfluorenyl‐2,7‐diyl)‐co‐(4,4′‐(N‐(4‐sec‐butyl‐phenyl)diphenylamine) (TFB) was purchased from American Dye Source inc. Trimethylaluminum (TMA, min. 98%) was purchased from Strem Chemicals, Inc. All chemicals were used without further purification.

### Perovskite VCSEL Fabrication

The commercial DBR (552FDN50, Knight Optical (UK) Ltd) was first ultrasonically cleaned with detergent, acetone, and isopropanol in turn for 10 min. The LiF was then thermally deposited onto the cleaned DBR in a PVD chamber with a rate of 0.5 A s^−1^. Afterward, the perovskite solution with a stoichiometry of CsPbBr_3_(BABr)_0.4_ was spin‐coated on top of LiF thin film and then annealed at 150 °C for 10 min. More details about perovskite thin film fabrication can be found in the previous paper^[^ ^21^ ^]^. To fabricate the VCSEL, the DBR/LiF/CsPbBr_3_ thin film with substrate sizes of 9 mm × 9 mm and 16 mm × 16 mm were placed face to face in a hot embossing machine^20^. The lamination process was conducted sequentially: 1) by heating up the substrates to 150 °C in N2 atmosphere; 2) by applying the force of 13500 N (equivalent to 166 MPa) for 5 min; 3) by releasing the force and cooling down the substrate to 50 °C. To distribute the force homogeneously onto the DBRs during lamination, two aluminum foils were placed between the DBR and embossing plates (top and bottom).

### Perovskite EAOPL Fabrication

First, 150 nm of Al_2_O_3_ was deposited via ALD onto the cleaned DBR. The LED consisting of 10 nm‐thick‐ITO, 30 nm‐thick‐ZnO, 5 nm‐thick‐Al_2_O_3_, 90 nm‐thick‐CsPbBr_3_, 30 nm‐thick‐TFB, 10 nm‐thick‐Al_2_O_3_, 10 nm‐thick‐ITO, and 200 nm‐thick‐Au grids (0.2 mm width) were then constructed on the same DBR substrate. The active area of LED is 3.5 × 3 mm with four sub‐cells on a single substrate. More details about LED fabrication can be found in the previous paper.^[^ ^15^ ^]^ Afterward, LiF was thermally evaporated as the cavity spacer, and a shadow mask was used during the LiF deposition to expose the ITO/Au contacts. To realize the lower lasing threshold for the EAOPL device, the thickness of LiF needs to be varied to locate the cavity mode to the wavelength at best optical gain. Finally, 100 nm‐thick‐Ag was thermally evaporated as the reflector to complete the EAOPL device. One of the sub‐cells was shadowed as the cavity‐free reference device while depositing Ag.

### Lasing Measurement

To pump the VCSEL or EAOPL device, a nanosecond pulsed laser (INNOLAS, MOPA 25, 355 nm, 1 ns) or a CW laser diode (Thorlabs, L405G1, 405 nm, 1 W) operated under QCW mode was used. The emission spectra were recorded using a SpectraPro HRS‐500 spectrometer equipped with a Princeton Instruments PIXIS:400BR CCD. The laser power was tuned by changing the position of variable‐neutral density filters (ND filters) and recorded in real time using a LabMax TOP (Coherent) power meter. A Keithley 2450 SMU was utilized to apply the DC voltage bias to the EAOPL device. The ND filters, power meter, optical shutter, SMU, and CCD were simultaneously controlled by a LabVIEW program. All the measurements were conducted in ambient air. Details on simultaneously pumping the CsPbBr_3_ LED with a pulsed/CW laser and a DC voltage can be referred to the previous publication.^[^ ^15^ ^]^

### Transient PL Measurement

The PL transient upon the excitation of INNOLAS pulsed laser or Thorlabs CW laser diode was measured in the same setup mentioned above for lasing measurement. A fast Si photodiode (Thorlabs, DET10A2) with a rise time of 1 ns was used to collect back emission from the VCSEL while collecting the front emission by the CCD. The photovoltage of the photodiode was then sent to an oscilloscope (Rohde&Schwarz, RTM2102) with the termination of 50 Ω and 1 MΩ for the excitation by nanosecond laser and QCW laser, respectively.

### Angle‐Resolved PL Measurement

To acquire the angle‐resolved PL, the VCSEL or EAOPL device was mounted in the center of the goniometer, and the CryLas pulsed laser (FTSS‐355‐Q, 1.3 ns, 355 nm) or the Thorlabs CW laser diode was fixed at the arm of the goniometer. The USB 2000+ Ocean Optics spectrometer was used to collect the emission spectra. The laser was normally incident onto the VCSEL or EAOPL device from the DBR side, and the emission was collected from the other side of the VCSEL or EAOPL device. A LabVIEW program was used to simultaneously control the movement of the goniometer, spectrometer, and SMU. All the measurements were conducted in the air.

### Fs‐TA Spectroscopy

The transient absorption was conducted on a custom‐built setup equipped with a femtosecond laser source (PHAROS, Light conversion) and an optical parametric amplifier (Orpheus, Light conversion). The fs‐TA data was collected via exciting the glass/CsPbBr_3_ sample with a 260 fs and 405 nm laser, and the measurements were all conducted under vacuum. More details about the fs‐TA setup can be found in the previous paper.^[^ ^22^ ^]^

### Transmittance Spectra

The VCSELs' transmittance spectra were recorded using a PerkinElmer Spectrophotometer (Lambda 1050 UV/Vis/NIR) equipped with an integrating sphere.

## Conflict of Interest

The authors declare no conflict of interest.

## Supporting information



Supporting Information

Supplementary Video 1

Supplementary Video 2

## Data Availability

The data that support the findings of this study are available from the corresponding author upon reasonable request.
