# Supplementary material for: Electrically‐Switchable Gain in Optically Pumped CsPbBr3 Lasers With Low Threshold at Nanosecond Pumping
Source: Small. 2025 Feb 24;21(13):2411935. doi: 10.1002/smll.202411935 (PMC11962692; doi:10.1002/smll.202411935)
Supplement: Supplementary file 1 — Supporting Information [file SMLL-21-2411935-s002.pdf]

NANO · MICRO
small

Supporting Information

for *Small*, DOI 10.1002/smll.202411935

Electrically-Switchable Gain in Optically Pumped $CsPbBr_3$ Lasers With Low Threshold at Nanosecond Pumping

Yang Li, Shangpu Liu, Thomas Feeney, Julie Roger, Mohammad Gholipoor, Hang Hu, Dewei Zhao, Ian Howard, Felix Deschler*, Uli Lemmer* and Ulrich W. Paetzold**

Supporting Information

Electrically-switchable gain in optically pumped $CsPbBr_3$ lasers with low threshold at nanosecond pumping

Yang Li, Shangpu Liu, Thomas Feeney, Julie Roger, Mohammad Gholipoor, Hang Hu, Dewei Zhao, Ian Howard, Felix Deschler*, Uli Lemmer*, Ulrich W. Paetzold**

Y. Li, D. Zhao
College of Materials Science and Engineering & Engineering Research Center of Alternative Energy Materials and Devices, Ministry of Education, Sichuan University, Chengdu, 610065, China

Y. Li, S. Liu, T. Feeney, J. Roger, M. Gholipoor, H. Hu, I. Howard, U. W. Paetzold
Institute of Microstructure Technology, Karlsruhe Institute of Technology, Hermann-von-Helmholtz-Platz 1, 76344 Eggenstein-Leopoldshafen, Germany.

Y. Li, T. Feeney, J. Roger, M. Gholipoor, H. Hu, U. Lemmer, U. W. Paetzold
Light Technology Institute, Karlsruhe Institute of Technology, Engesserstrasse 13, 76131 Karlsruhe, Germany.

S. Liu, F. Deschler
Physikalisch-Chemisches Institut, Universität Heidelberg, Im Neuenheimer Feld 229, 69120 Heidelberg, Germany.

E-mail: yangli2024@scu.edu.cn, felix.deschler@pci.uni-heidelberg.de, ulrich.lemmer@kit.edu, ulrich.paetzold@kit.edu

Table of Contents

Figure S1 Transmittance spectra of VCSEL·····3

Figure S2 Cavity-photon dispersion of VCSEL·····4

Figure S3 Transient optical gain spectra·····5

Figure S4 Pump pulse length dependent carrier transients·····7

Figure S5 Emission feature of VCSEL under CW operation at room temperature·····9

Figure S6 Emission transient of QCW laser·····11

Figure S7 Emission feature of $CsPbBr_3$ thin film under QCW laser excitation·····12

Figure S8 Emission feature of VCSEL under CW operation at 80 K·····13

Figure S9 Effect of k_2 on CW lasing·····14

Figure S10 Cross-sectional SEM image of EAOPL device·····15

Figure S11 DC bias enhances PL lifetime of $CsPbBr_3$ thin film·····16

Figure S12 Angle resolved emission of $CsPbBr_3$ thin film·····17

Figure S13 Effect of DC bias on VCSEL emission under CW operation·····18

Table S1 Summary of the reported lasing/ASE thresholds·····19

Table S2 Summary of the FWHM for $CsPbBr_3$ thin film and device·····23

Video S1 VCSEL beam projection and stability·····20

Video S2 Electrically switchable lasing in a perovskite VCSEL·····24

Reference·····25

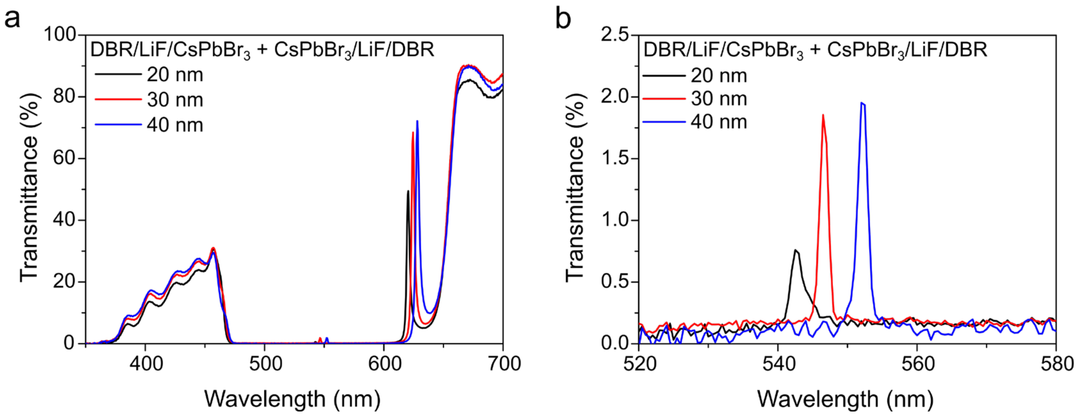

Figure S1. Transmittance spectra of VCSEL. a-b) Transmittance spectra of VCSELs laminated from two DBR/LiF/$CsPbBr_3$ thin films with a series of LiF thickness in the wavelength range of a) 350 nm to 700 nm and b) 520 nm to 580 nm. The resonance signature within the stopband of DBR demonstrates the homogenous cavity within the UV-Vis absorption measurement area (~3 mm × 3 mm).

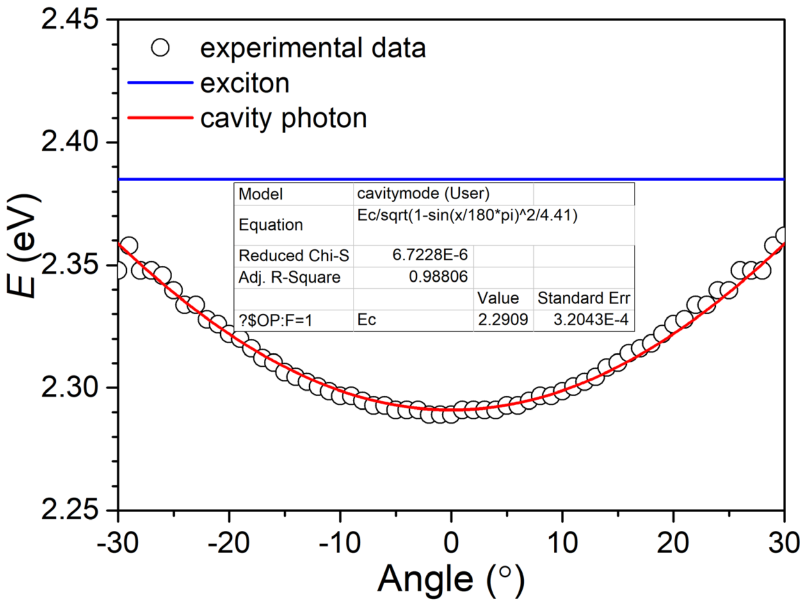

Figure S2. Cavity-photon dispersion of VCSEL. The dispersion extracted from the peak positions (black circles) of angle-resolved emission spectra (spontaneous emission, below threshold) shown in Figure 1d.

The cavity photon (E_{cav}) and exciton-polariton ($E_{LP,UP(k_{\|})}$) are given by

$$E_{cav} = E_{ph}(0)(1-\frac{\sin^2\theta}{n_c^2})^{-1/2}$$

$$E_{LP,UP(k_{\|})} = \frac{1}{2}[E_{exc} + E_{cav} \pm \sqrt{4g_0^2 + (E_{exc} - E_{cav})^2}]$$

Where $E_{ph}(0)$ is the cavity resonance mode, n_c is the effective refractive index (estimated as 2.1), E_{exc} is the exciton energy (estimated as 2.385 eV), ($2g_0$) is the Rabi splitting. The experimental data fit the cavity photon dispersion (red curve) well, producing a cavity resonance of 2.29 eV (541 nm), but failed to fit the exciton-polariton dispersion. These results suggest the absence of exciton-polariton in the laminated $CsPbBr_3$ VCSEL.

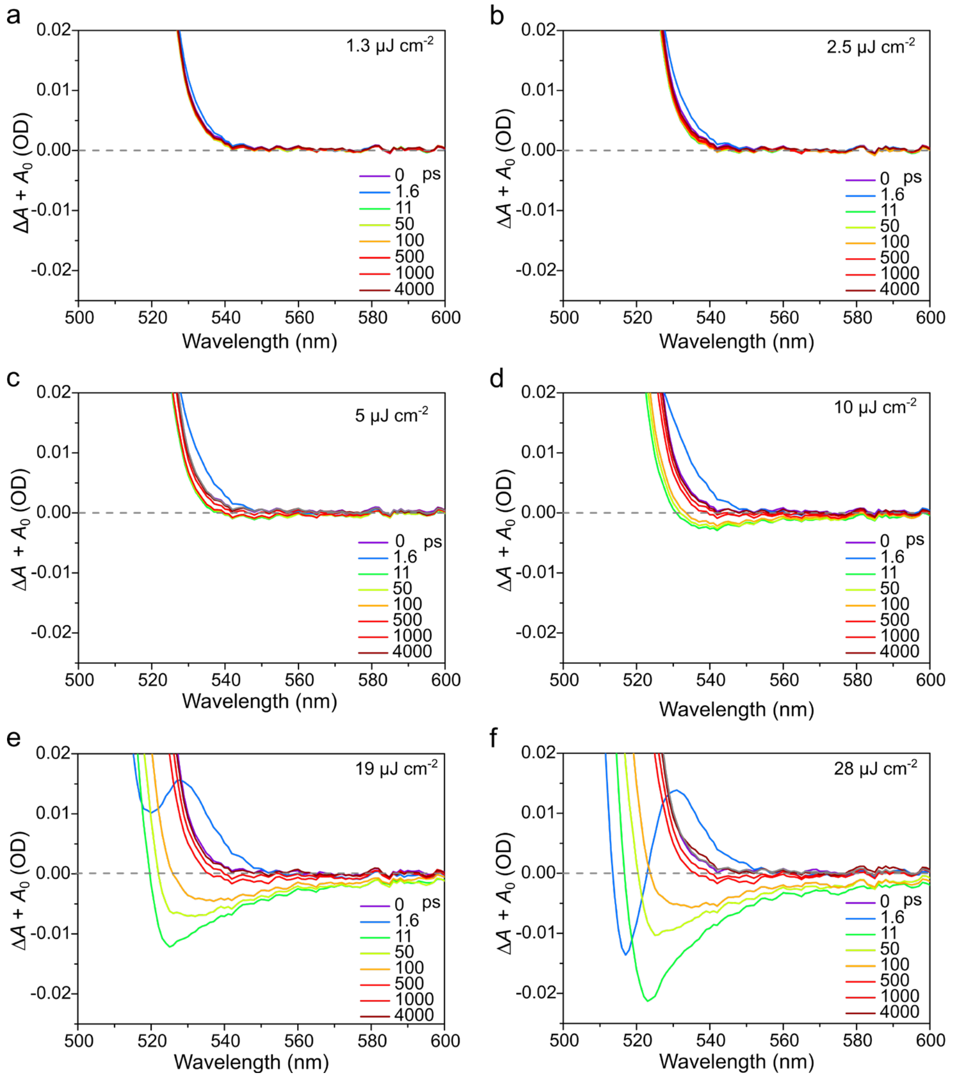

Figure S3. Transient optical gain spectra. a-f) Transient absorbance of $CsPbBr_3$ thin film following fs pump laser excitation derived from the fs-TA spectra, which are acquired by a 405 nm (260 fs) laser excitation at a series of pump energy densities from 1.3 to 28 μJ cm^{-2}.

In fs-TA pump-probe experiments, the absorbance changes, $\Delta A(\lambda,t)$, represent the difference in absorbance of the sample to the probe beam when the pump beam is turned ON and OFF. The latter are the linear absorption of the sample (A_0). Then we have $\Delta A(\lambda,t) = A_{pump\_on} - A_{pump\_off} = A(\lambda,t) - A_0$. The appearance of negative absorption while the pump beam is turned ON ($A = \Delta A + A_0 \leq 0$) indicates the onset of optical gain, which starts ~10 μJ cm^{-2} under fs laser excitation.

We note that the best gain for transient gain spectrum (525 nm), ASE spectrum (536 nm) and threshold spectrum (540 nm) shown in Figure 1f were different. It can be

explained as: (1) The best transient gain shifted to longer wavelength along with the time delay evolution upon fs laser excitation. This causes the redshift of ASE spectrum compared to transient gain spectrum. (2) Both transient gain spectrum and ASE spectrum were measured with the cavity free thin films, which generally considered as the single trip gain. However, the threshold spectrum was measured with VCSEL, where the photons will travel back and forth between the reflectors (multiple trip gain). The re-absorption process can lead to a preferential amplification of longer wavelength light. Hence, the best threshold spectrum was expected to be redshift compared to ASE spectrum.

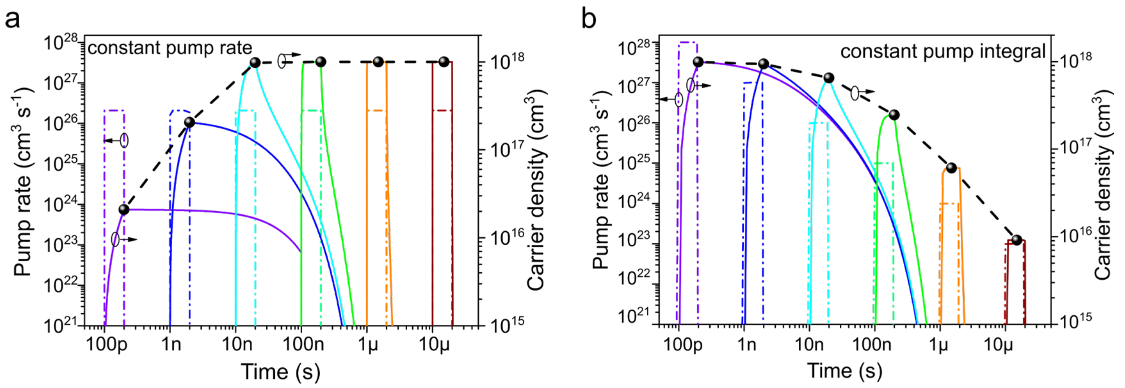

Figure S4. Pump pulse length dependent carrier transients. a-b) Simulated transient carrier densities under a) constant pump rate (2.1×10^{26} cm^{-3} s^{-1}) and b) constant pump flux (10^{18} cm^{-3}) versus pump pulse length. The black spheres indicate the maximum carrier densities.

To understand the dependence of reported lasing (power and energy) thresholds with pump pulse lengths, the transient carrier densities were simulated via numerically solving the rate equation $dn/dt = G(t) - k_1n - k_2n^2 - k_3n^3$, where k_1 (10^7 s^{-1}), k_2 (10^{-10} cm^3 s^{-1}), k_3 (10^{-28} cm^6 s^{-1}) and G(t) are 1st, 2nd, 3rd order rate coefficients and square wave generation function, respectively. The simulation is conducted by fixing G(t) or the integral of G(t) over time. Under constant pumping rate (Figure S4a), the carrier population within the pump pulse continuously increases with pulse length once steady-state condition is established, leading to the increase in maximum carrier density. Hence, to generate same carrier densities, lower pump power is required for longer pulse than shorter pulse. Hence, power density thresholds apparently decrease with increasing pump pulse length, as shown in Figure 2a (right panel). In contrast, under constant pump flux (Figure S4b), the maximum carrier densities remain roughly same when the pump pulse length is less than 1 ns, and further decrease significantly with increasing pump pulse length. This generally agrees with the energy density threshold as shown in Figure 2a (left panel).

Hence, if the pump pulses are short (much less than 1 ns) and appear "instantaneous" enough to the system, the pulse energy needed for lasing remains roughly the same. Therefore, it makes sense to use energy density for lasing threshold. If the pump pulse length is large (greater than 1 ns) and the steady-state condition is

established within the pump pulse, the pulse power needed for lasing remains roughly the same. Thus, it makes sense to use power density for lasing threshold.

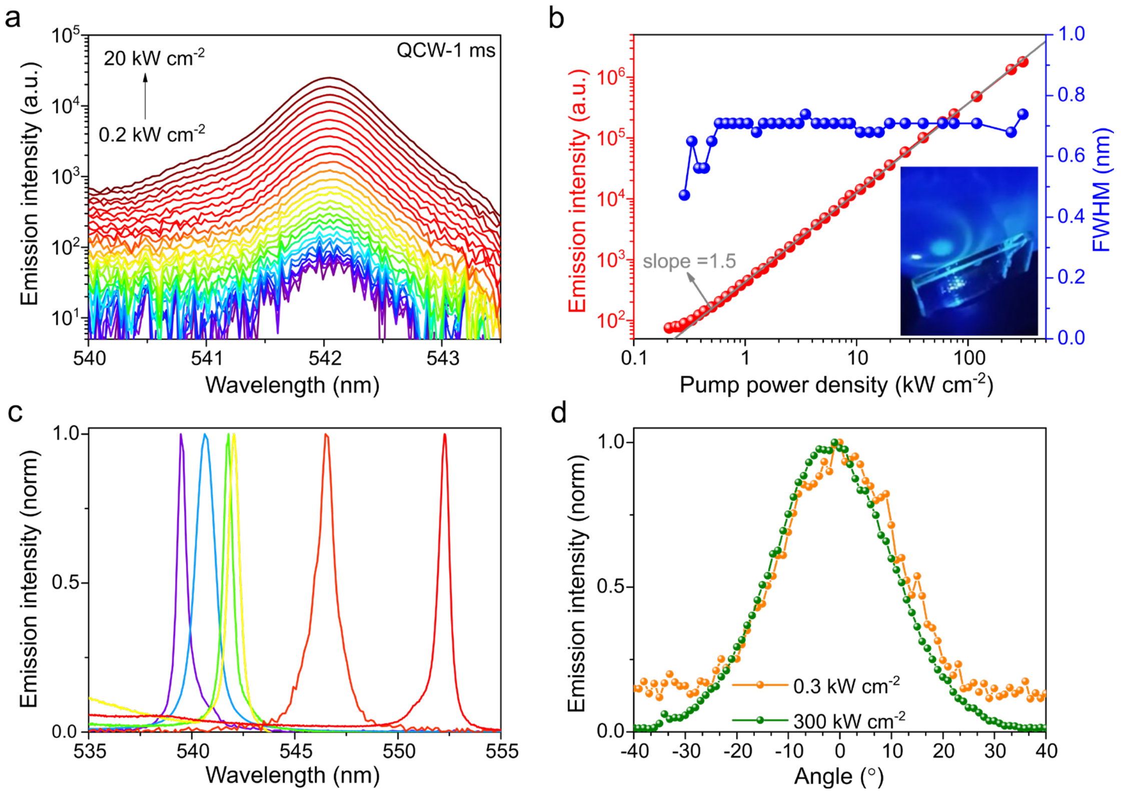

Figure S5. Emission feature of VCSEL under CW operation at room temperature. a) Emission spectra of VCSEL pumped by a (405 nm) QCW laser with 1 ms pump length at a series of pump power densities. b) Plots of emission intensity and FWHM vary with pump power densities. The dashed gray line is the linear fit of the light-in light-out curve. The inset shows the far-field image of the emission beam from VCSEL (green) and QCW pump laser (blue, incidence at 45º), where a card is placed in front of VCSEL at ~3 cm. c) Normalized emission spectra for a series of VCSELs. d) Plots of emission intensity at 0.3 kW cm^{-2} and 300 kW cm^{-2} vary with angle.

Several laser-like features under QCW excitation are visible: (1) narrow emission linewidth (Figure S5a, FWHM of 0.7 nm), (2) circular emission beam (Figure S5b, inset), and (3) tunable emission peak by resonator (Figure S5c, via LiF thickness). However, both output power and FHWM show no clear threshold behavior. As shown in Figure S5d, the almost unchanged beam divergence for a wide range of pump powers (0.3 – 300 kW cm^{-2}) further excludes the existence of threshold-less lasing behavior. Hence, the current laminated $CsPbBr_3$ VCSEL didn't support room temperature CW lasing. To avoid confusion, we would also like to point out that the features of (1) to

(3) cannot distinguish between cavity mode emission and laser emission, and they cannot be used to identify laser emission.

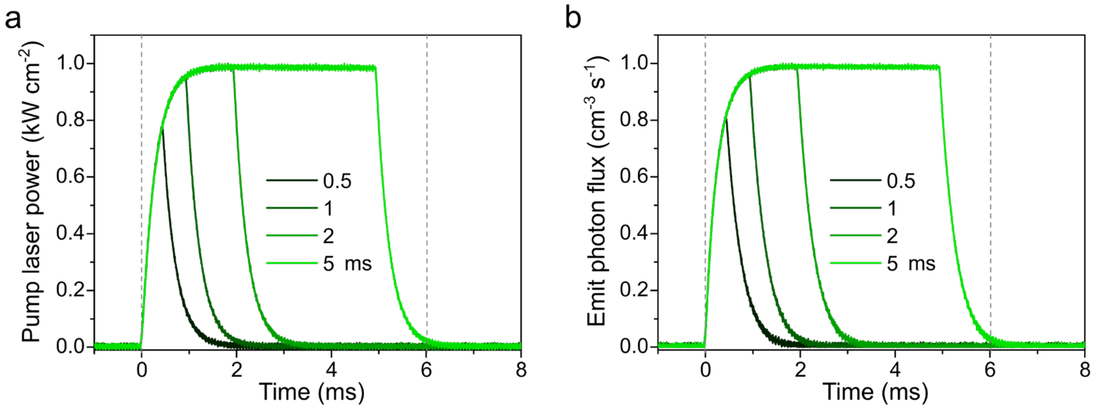

Figure S6. Emission transient of QCW laser. a) QCW laser intensity varies with pump pulse lengths. The laser power is fixed at 1 kW cm^{-2} while modulating the CW laser into pulse mode. b) Simulated emission transient at power density of 1 kW cm^{-2} versus pump pulse length. The simulation is based on the rate equations $dn/dt = G(t) - k_1n - k_2n^2 - k_3n^3$ and $dn_p/dt = k_2n^2$, where k_1 (10^7 s^{-1}), k_2 (10^{-10} cm^3 s^{-1}), k_3 (10^{-28} cm^6 s^{-1}) and G are 1st, 2nd, 3rd rate coefficient and pump rate. G sets as 1 kW cm^{-2} for the simulation, corresponding to a pump rate of 2.3×10^{26} cm^{-3} s^{-1}.

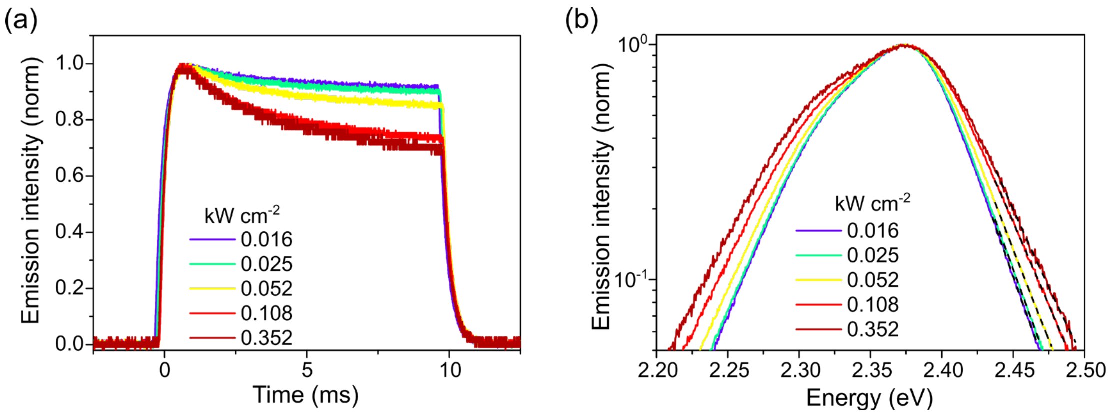

Figure S7. Emission feature of $CsPbBr_3$ thin film under QCW laser excitation. a) Normalized transient emission intensity and b) normalized steady-state emission spectra of glass/$CsPbBr_3$ thin film pumped by a QCW laser with a pulse length of 10 ms at a series of power densities. The electron-hole plasma temperatures were extracted by fitting (black dashed lines in Fig S7b) the high energy till of PL spectra with $Ae^{-E/(kT)}$ exponential function[1,2]. Upon increasing the pump power of CW laser from 0.016 kW cm^{-2} to 0.352 kW cm^{-2}, the temperature increases from 290 K to 357 K indicating the serious heating effect by the CW pump laser.

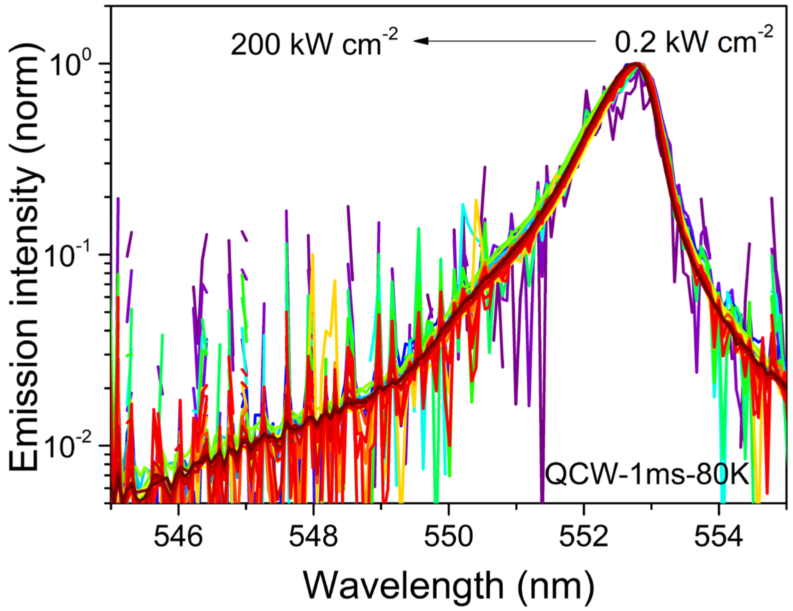

Figure S8. Emission feature of VCSEL under CW operation at 80 K. Normalized emission spectra of VCSEL pumped by the QCW laser under a series of pump power densities at 80 K. Upon increasing pump power densities from 0.2 kW cm^{-2} to 200 kW cm^{-2}, the normalized spectra remain almost unchanged, and no clear spectral narrowing is visible, suggesting the absence of CW lasing at 80 K.

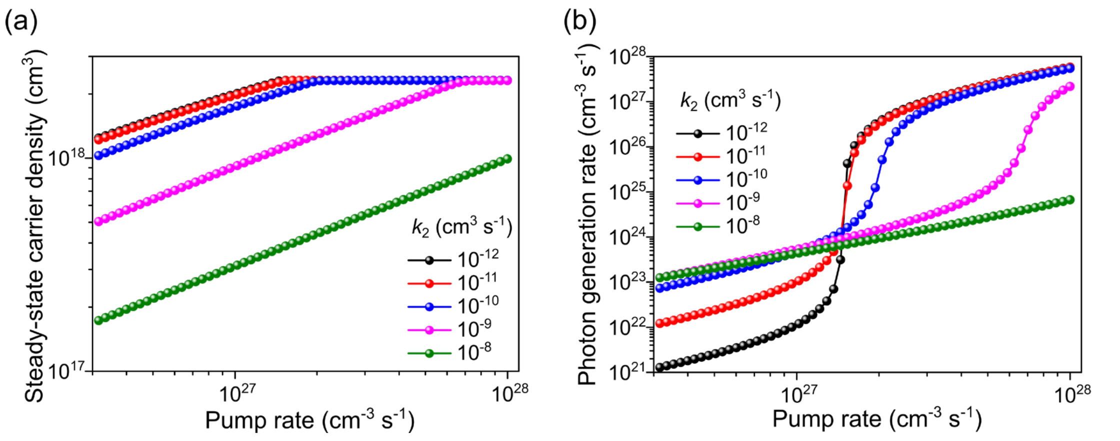

Figure S9. Effect of k_2 on CW lasing. a-b) Simulated a) steady-state carrier density and b) photon generation rate versus pump rate by numerically solving the laser diode rate equations with varying k_2.

The following laser diode rate equations were used for the simulation:

$$\frac{dn}{dt} = G - k_1 n + k_2 n^2 + k_3 n^3 + (n - n_{\mathrm{tr}})\sigma_{\mathrm{st}} v_{\mathrm{g}} n_{\mathrm{p}}$$

$$\frac{dn_{\mathrm{p}}}{dt} = \Gamma \beta_{sp} k_2 n^2 + \Gamma (n - n_{\mathrm{tr}})\sigma_{\mathrm{st}} v_{\mathrm{g}} n_{\mathrm{p}} - \frac{n_{\mathrm{p}}}{\tau_p}$$

where n, n_{p} G, k_1 (10^8 s^{-1}), k_2, k_3 (10^{-28} cm^6 s^{-1}), n_{tr} (10^{18} cm^{-3}), σ_{st} (10^{-16} cm^2), ν_{g} (5.6×10^9 cm s^{-1}), Γ (0.68), β_{st} (10^{-3}), and τ_{p} (2.6 ps) are the carrier density, photon density, pump rate, 1st rate coefficient, 2nd rate coefficient, 3rd rate coefficients, transparency carrier density, stimulated emission cross-section, group velocity, confinement factor, spontaneous emission coupling factor, and photon lifetime, respectively[3]. As shown in Fig S9b, increasing the 2nd rate coefficient from 10^{-12}cm^3 s^{-1} to 10^{-8} cm^3 s^{-1} leads to a higher photon generate rate below lasing threshold. However, a much higher radiative rate (>10^{-10} cm^3 s^{-1}) cause the lower steady-state carrier density (Fig S9a), results in a higher lasing threshold (Fig S9b). Hence, the 2nd radiative rate affects the stimulated emission rate and population inversion in opposite ways. A higher radiative rate does not necessarily benefit the CW lasing.

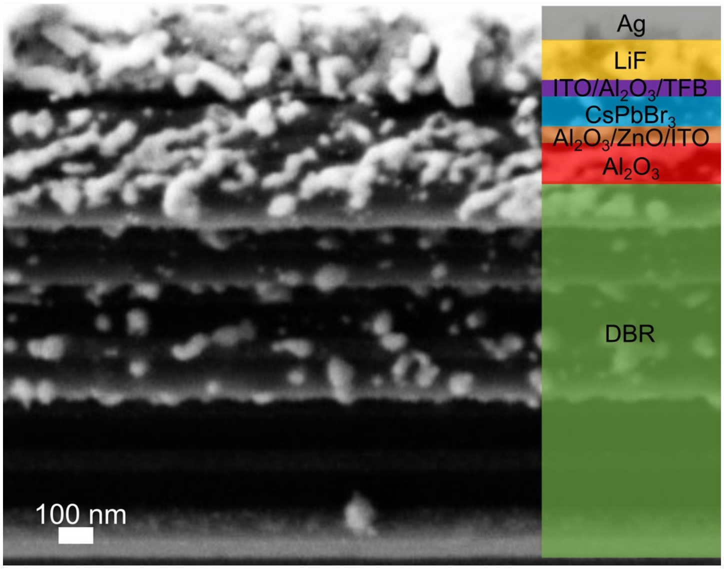

Figure S10. Cross-sectional SEM image of the EAOPL device. The functional layers were highlighted in different colors to serve as a guide for the eye, especially since some of these layers are too thin to be easily recognized. The full layer stack is around 1.8 µm.

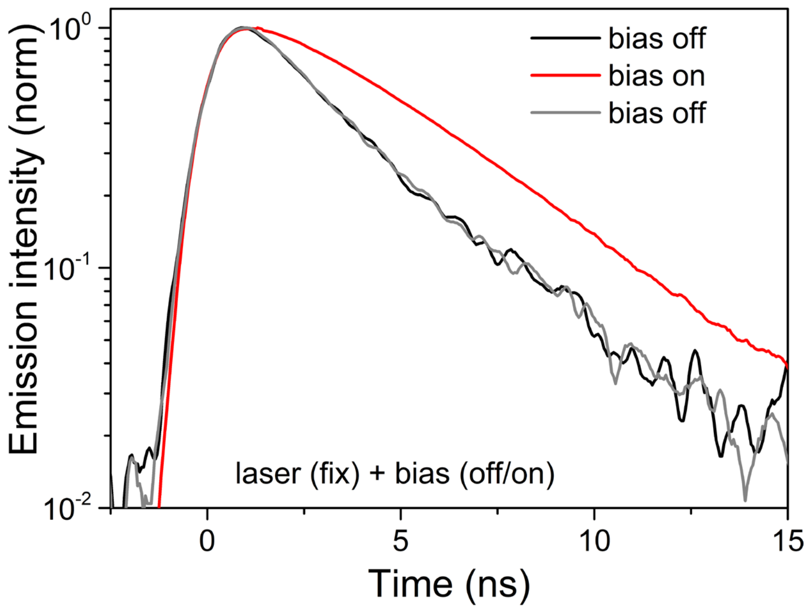

Figure S11. DC bias enhances PL lifetime of $CsPbBr_3$ thin film. Comparison of TPRLs for open cavity device between switching on and off 4 V DC bias while keeping the power of pump laser at $0.6P_{th}$. The TRPLs are collected using a fast Si photodetector connected to an oscilloscope, and the pump laser (355 nm) pulse length is 1 ns.

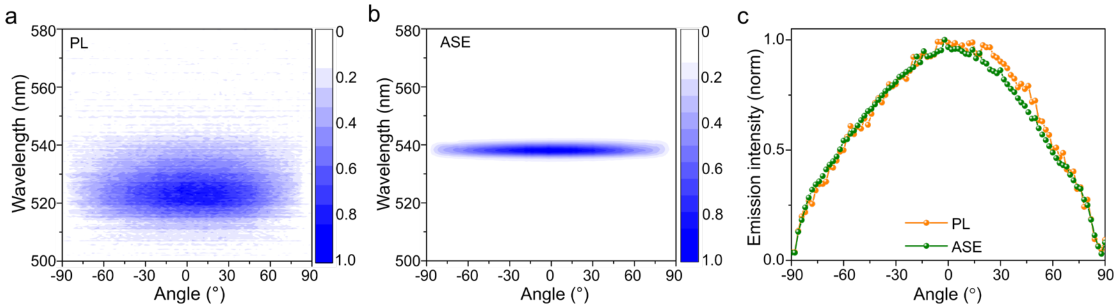

Figure S12. Angle resolved emission of $CsPbBr_3$ thin film. a-b) Angle dependent a) PL and b) ASE of $CsPbBr_3$ thin film deposited on glass substrate. c) Plots of emission intensity versus angle.

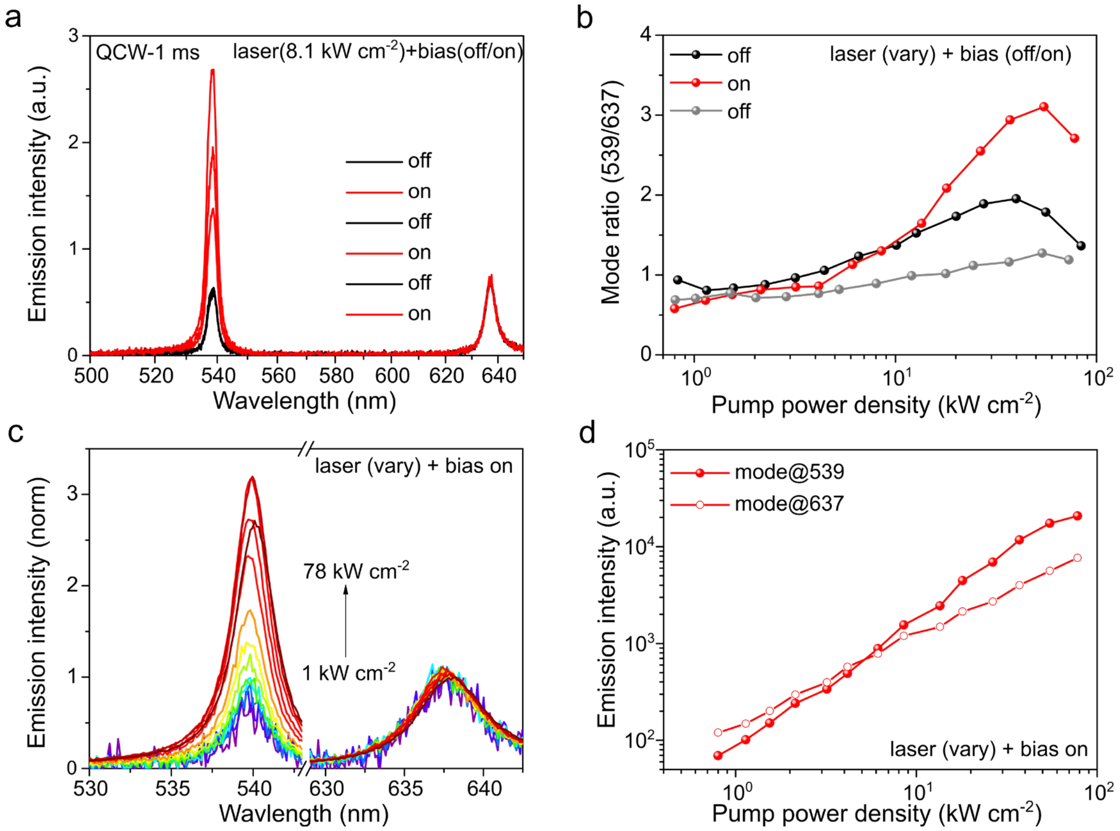

Figure S13. Effect of DC bias on VCSEL emission under CW operation. a) Emission spectra of EAOPL device pumped by a QCW laser (1 ms pulse length) at a 4 V DC bias. The DC bias is switched between off and on while the power of the pump laser is kept at 8.1 kW cm^{-2}. b) Mode intensity ratio versus pump power density with or without 4 V bias. c) Emission spectra under a series of pump power densities while keeping the 4 V bias on. The spectra are normalized to the intensity at cavity mode of 637 nm for better comparison. d) Plots of emission intensity vary with pump power densities while keeping the 4 V bias on.

Table S1. Summary of the reported lasing/ASE thresholds

Materials	Cavity	Pump (nm)	Emission (nm)	Pulse (ns)	Threshold (uJ cm^{-2})	Threshold (kW cm^{-2})	T (K)	FWHM (nm)	reference
$MAPbI_3$ thin film	DFB	445	790	20	--	5	160	1	*Nano Lett.* **2016**, *16*, 7, 4624
$MAPbI_3$ thin film	DFB	445	785	CW	--	17	160	0.25	*Nat Photon.* **2017**, *11*, 784
$Cs_{0.1}(MA_{0.17}FA_{0.83})_{0.9}Pb_{0.84}(I_{0.84}Br_{0.16})_{2.68}$ thin film	ASE	532	790	CW	--	0.387	80	--	*Nat. Commun.* **2019**, *10*, 988
N2F8 quasi-2D thin film	DFB	488	555	CW	--	0.059	RT	0.8	*Nature* **2020**, *585*, 53
P2F8 quasi-2D thin film	DFB	488	553	CW	--	0.045	RT	1	
$CsPb(I_xBr_{1-x})_3$-$Zn(Ac)_2$ thin film	DFB	450	714.3	CW	--	0.25	110	0.125	*Adv. Mater.* **2023**, *35*, 2303144
$(NMA)_2FA_{n-1}PbnI_{3n+1}$ thin film	DFB	450	809	200000	--	0.9116	110	0.17	*Adv. Funct. Mater.* **2023**, 2303900
$Cs_{0.1}FA_{0.9}PbI_{2.855}Br_{0.145}$ LED	ASE	447	790	1000	--	3.8	70	--	*Nat. Photon.* **2024**, *18*, 132
$CH_3NH_3PbBr_3$ single crystal thin film	DBR-DBR	397	544-554	2E-4	26	130000	RT	1	*Appl. Phys. Lett.* **2016**, *108*, 261105
$CH_3NH_3PbI_3$ thin film	DBR-DBR	532	778.4	0.34	7.6	22.35	RT	0.24	*Adv. Mater.* **2017**, *29*, 1604781
				5	114	22.8			
$CsPbBr_3$ QD thin film	DBR-DBR	400	505	1E-4	11	1.1E5	RT	0.6	*Adv. Funct. Mater.* **2017**, *27*, 1605088
				5	900	180		--	
$CsPb(BrCl)_3$ QD thin film			470	1E-4	25.5	255000		--	
$CsPb(BrI)_3$ QD thin film			595	1E-4	19	190000		--	

$CsPbBr_3$ QD thin film	DBR-DBR	400	522	5E-5	0.39	7800	RT	0.9	*ACS Photonics* **2017**, *4*, 2281
		355	523	5	98	19.6		--	
$FAPbBr_3$ thin film	DBR-DBR	355	552.4	0.34	18.3	53.8	RT	0.28	*ACS Photonics* **2017**, *4*, 2486
$Cs_{0.17}FA_{0.83}PbBr_3$ thin film	DBR-DBR	355	552.6	0.34	13.5	39.71	RT	0.4	*Optica* **2018**, *5*, 1141
$CsPbBr_3$ thin film	DBR-DBR	400	542	1.5E-4	1.7	11333.3	RT	0.51	*ACS Appl. Mater. Interfaces* **2018**, *10*, 40661
$(PEA)_2Cs_{n-1}Pb_nBr_{3n+1}$ microcrystals	DBR-Ag	355	532	8	500	62.5	RT	0.8	*Appl. Phys. Lett.* **2019**, *114*, 131107
$CsPbBr_3$ thin film	DBR-DBR	355	538	0.3	2.2	7.33	RT	0.07	*Adv. Mater.* **2019**, *31*, 1903717
$CH_3NH_3PbCl_3$ single crystal thin film	DBR-DBR	355	414-435	8	211	26.375	RT	0.38	*Opt. Mater.* **2020**, *107*, 110130
$BA_2Cs_5Pb_6Cl_{19}$ thin films	DBR-DBR	337	425	2.5E-4	6.5	26000	RT	1.35	*Adv. Mater.* **2021**, *33*, 2006697
$CsPbCl_{1.5}Br_{1.5}$ thin films	DBR-DBR	355	479	8E-5	9.2	115000	RT	0.41	*Nano Lett.* **2022**, *22*, 3, 1338
Quasi-2D/3D planar heterostructure	DBR-Ag	355	538	1.1	46	41.82	RT	1.3	*Adv. Funct. Mater.* **2022**, *32*, 2200772
$MAPbI_3$ thin films	DFB	532	779	1	0.32	0.32	RT	2.2	*Adv. Mater.* **2016**, *28*, 923
			784	1	0.54	0.54		2.1	
			793	1	2.11	2.11		1.4	
$MAPbI_3$ thin films	DFB	515	--	2E-4	4	20000	RT	--	*Opt. Express* **2016**, *24*, 23677
		355	784	0.91	110	120.8		0.4	
$MAPbI_3$ thin films	DFB	532	786.5	1	120	120	RT	0.2	*Appl. Phys. Lett.* **2016**, *109*, 141106
$CsPbBrI_2$-PEO thin films	DFB	355	654	9E-2	33	366.7	RT	4.9	*Opt. Express* **2017**, *25*, A1154

$MAPbBr_3$ thin films	DFB	355	550	1	6	6	RT	<1	*Sci. Rep.* **2017**, *7*, 11727
$MAPbBr_3$ thin films	DFB	532	555	0.3	3.4	11.3	RT	0.14	*Adv. Mater. Technol.* **2018**, *3*, 1700253
$MAPbI_3$ thin films	DFB	532	784	1	235	235	RT	0.4	*Opt. Express* **2018**, *26*, A144
$MAPbI_3$ thin films	DFB	--	798	0.8	7	8.8	RT	0.8	*ACS Photonics* **2019**, *6*, 460
$CsPbBr_3$ thin film	DFB	355	539	0.3	7.2	24	RT	0.14	*Adv. Mater.* **2019**, *31*, 1903717
$Cs_xMA_{1-x}Pb(I_{1-x}Br_x)_3$ and $FPMAI_{1-x}Br_x$ thin film	DFB	355	500-800	0.04	4	100	RT	0.65	*ACS Photonics* **2019**, *6*, 3331
$MAPbI_3$:BAI thin films	DFB	470	790	0.02	6	300	RT	1.19	*Adv. Opt. Mater.* **2020**, *8*, 1901297
N2F8 quasi-2D thin film	DFB	337	559	3	4.7	1.57	RT	0.45	*Nature* **2020**, *585*, 53
P2F8 quasi-2D thin film			552	3	32.8	10.93			
$MAPbI_3$ thin film	DFB	532	791	4E-5	2.75	68750	RT	0.8	*ACS Photonics* **2021**, *8*, 2548
TOL-N2F4 thin film	DBR-DBR	355	546	10	29	2.9	RT	2.6-1.4	*J. Phys. Chem. Lett.* **2023**, *14*, 2493
EA-N2F4 thin film			544	10	14	1.4		1.7-0.8	
$CsPbBr_3$	DBR-DBR +microlens	450	530.6	8E-5	61	762500	RT	0.06	*Nano Lett.* **2024**, *24*, 4, 1406
$(FAPbI_3)_{0.95}(MAPbBr_3)_{0.05}$ thin film	DFB	532	787-830	2.3	130	56.52	RT	0.075	*Adv. Opt. Mater.* **2024**, *12*, 2302496
$CsPbBr_3$:TPPO thin films	SiO_2-SiO_2	400	532	3.5E-5	13	371428	RT	0.24	*Adv. Mater.* **2023**, *35*, 2306102
4-AMP DJ thin film	DFB	355	532	0.5	8.9	17.8	RT	0.018	*Sci. Adv.* **2023**, *9*, eadj3476
DBA DJ thin film			535	0.5	5.5	11		0.021	

$NMA_2FA_8Pb_8Br_{26}$ quasi-2D thin film	DFB	337	537	0.8	1	1.25	RT	1	*Adv. Funct. Mater.* **2023**, *33*, 2301794
$CsPbBr_3$ NCs film	DBR-DBR	400	527	3.5E-5	22	628571	RT	0.49	*Nanophotonics* **2023**, *12*, 2133
$FAPbBr_3$ thin film	DFB	337	555	3.5	22.6	6.46	RT	0.6	*Nanotechnology*, **2023**, *34*, 175201
$CsPbBr_3$ thin film	DBR-DBR	355	540	1	1.3	1.3	RT	0.39	**This work**

Table S2. Summary of the FWHM for $CsPbBr_3$ thin film and device

FWHM	$CsPbBr_3$ thin film	Laminate VCSEL	EAOPL device
Below threshold	20 nm (PL)	1.2 nm (cavity emission)	3.4 nm (cavity emission)
Over threshold	3.2 nm (ASE)	0.39 nm (Lasing)	1.5 nm (Lasing)

Supplementary videos are available online that show:

Video S1. VCSEL Beam Projection and Stability. This video demonstrates the projection of a VCSEL beam onto a card. The card is moved from the VCSEL surface to a distance of over 20 cm along the surface normal, and then back to the VCSEL surface. The pump laser is incident at 45° angle. This showcases the beam's stability and directionality.

Video S2. Electrically Switchable Lasing in a Perovskite VCSEL. The movie shows the appearance and disappearance of the green lasing spot as positive 4 V DC bias is toggled on and off. The pulsed laser power is maintained at $0.6P_{th}$ (60% of the purely optically pumped lasing threshold). The emission is stabilized for ~5 s after switching the bias.

Reference

[1] M. Cadelano, V. Sarritzu, N. Sestu, D. Marongiu, F. Chen, R. Piras, R. Corpino, C. M. Carbonaro, F. Quochi, M. Saba, A. Mura, G. Bongiovanni, *Adv. Optical Mater.* **2015**, *3*, 1557.

[2] K. Leo, W. W. Rühle, K. Ploog, *Phys. Rev. B*, **1988**, *38*, 1947.

[3] Y. Jia, R. A. Kerner, A. J. Grede, B. P. Rand, N. C. Giebink, *Adv. Optical Mater.* **2020**, *8*, 1901514.
